# The value of advanced practice nursing in Danish primary care: a case study of communicative and integrative mechanisms in community-based care

**DOI:** 10.1186/s12875-026-03207-7

**Published:** 2026-02-06

**Authors:** Charlotte Laubek, Janus Laust Thomsen

**Affiliations:** 1 Department of Culture and Learning, Aalborg University, Aalborg, Denmark; 2 Center for Almen Medicin, Det Sundhedsvidenskabelige Fakultet, Aalborg University, Aalborg, Denmark

**Keywords:** Advanced practice nursing, Chronic care model, Fundamentals of care, Interprofessional collaboration, Multimorbidity, Community-based healthcare.

## Abstract

**Background:**

The growing prevalence of multimorbidity and complex care needs places increasing demands on primary healthcare systems. In Denmark, Advanced Practice Nurses (APNs) have been introduced in municipal community-based healthcare to strengthen continuity, coordination, and patient-centered care. While international evidence demonstrates positive outcomes of APN-led care, limited research has explored *how* APNs create value in everyday practice.

**Aim:**

To explore how APNs contribute to communication, collaboration, and competence integration in complex, cross-sectoral care trajectories in a Danish municipality.

**Methods:**

A qualitative case study was conducted in Aalborg, the third-largest city in Denmark (224,537 inhabitants). Data comprised interviews involving 24 participants (patients, relatives, primary care professionals, and general practitioners) and a reflective workshop with six APNs and their leader (*n* = 7). Data were thematically analyzed and interpreted using the Chronic Care Model as a framework for integrated and people-centered care.

**Results:**

Two interrelated themes emerged. First, APNs acted as strategic communicators and accountable coordinators, facilitating dialogue, stabilizing fragmented trajectories, and enabling collective sensemaking across professional and organizational boundaries. Second, APNs demonstrated competence integration from fundamental to advanced nursing, combining relational engagement, fundamental care, and advanced clinical reasoning within the same encounter. By grounding interventions in patients’ perceived problems, APNs uncovered how fundamental needs often underpin complex trajectories. These findings extend theoretical understandings of care integration and empirically substantiate the Fundamentals of Care framework.

**Conclusion:**

APNs strengthen integrated, people-centered community-based primary care not only through advanced clinical expertise but by managing the communicative and relational infrastructure that sustains collaboration and continuity. This study contributes with process-oriented evidence addressing international knowledge gaps and demonstrates how local practice innovations, such as the *APN triage*, can operationalize global visions for integrated, equitable, and sustainable primary healthcare.

**Supplementary Information:**

The online version contains supplementary material available at https://doi.org/10.1186/s12875-026-03207-7.

## Introduction

The growing complexity of care in primary healthcare systems is driven by demographic change, rising multimorbidity, and fragmented service delivery [1–4]. In Denmark, a small group of patients with highly complex care needs account for a disproportionately large share of healthcare utilization [5]. The care needs of these patients typically involve multiple providers across sectors, leading to coordination challenges and risks of suboptimal outcomes [6].

In 2019, the Advanced Practice Nurse (APN) master’s degree program in advanced clinical nursing was introduced in Denmark in a collaboration between universities and municipalities [7]. Since then, APNs have become an integral part of e.g., municipal health services, which form the core of community-based healthcare in Denmark. Evaluations indicate that APNs strengthen the systematization of care [8, 9], act as clinical knowledge brokers, and contribute to reducing unplanned avoidable hospital readmissions while enhancing interdisciplinary collaboration [10, 11]. However, the Danish and Nordic literature on the subject generally remains descriptive or evaluative, focusing on competency development [7], organizational integration [10], or aggregate outcome metrics such as readmission rates [11].

While previous Scandinavian studies have examined APN role development [12, 13], educational frameworks, and patient satisfaction in primary care [14–18], research has been limited on exploring the embedded, everyday practices of APNs in a mature municipal implementation model. This study addresses this gap by investigating how APNs function as clinical coordinators, knowledge brokers, and relational anchors in cross-sectoral care for patients with highly complex care needs. By adopting a practice-based perspective from a Danish municipality, the study highlights the systemic impact of APNs on collaboration, continuity of care, and prevention.

Thus, the study addresses the following research question:

*How do Advanced Practice Nurses (APNs) create value for patients*,* relatives*,* municipal collaborators*,* and general practitioners in the coordination of complex care trajectories in community-based healthcare in Denmark?*

## State of the art

The World Health Organization (WHO) has repeatedly called for expanding nurses’ scope of practice, including the introduction of APNs, to strengthen primary care and promote equitable access to services in the light of an increasing number of patients with multimorbidity and chronic disease [19]. At the same time, WHO emphasizes that health systems must shift more care from hospitals to primary and community-based settings to address overcrowding, rising costs, and ensure sustainable universal health coverage [20]. Similarly, international case studies have demonstrated that “moving care from hospital into the community” is a key strategy to improve accessibility, continuity of care, and overall system performance [21].

International evidence consistently shows that APNs are well-positioned to meet these challenges. A recent umbrella review of 117 systematic reviews found that care delivered by APNs, nurse practitioners (NPs), and clinical nurse specialists (CNSs) is at least equivalent - and often superior - to physician-led care across outcomes such as clinical indicators, patient satisfaction, safety, and mortality. Importantly, no outcomes consistently favored usual care, underscoring the robustness of APN contributions across health systems [20].

To conceptualize and organize such complex, multi-level care, the Chronic Care Model (CCM) has often been applied in primary and community settings. An umbrella review of 26 systematic reviews showed that nursing processes aligned with CCM principles, particularly self-management support and nurse-led case management, are consistently associated with positive outcomes. However, the same review highlighted persistent gaps in *decision support* and *clinical information systems*, limiting the full realization of the potential of APNs in chronic care [22].

Recent Danish studies have also illustrated the value of APNs in community-based healthcare. A national scientific evaluation demonstrated that APNs provide systematic and holistic assessments, help prevent unplanned avoidable hospital admissions, coordinate interprofessional care pathways, and act as role models and capacity-builders for other nurses in municipal primary care. Crucially, professional autonomy was identified as a prerequisite for APNs to achieve full impact on patient safety, quality, and system efficiency [10].

Although APN-led models are also expanding in hospital settings [23], this study focuses deliberately on *primary and community-based care*, reflecting demographic and policy priorities in Denmark and internationally, where care for people with multimorbidity is increasingly shifting from hospitals to community-based care and general practice [20, 24].

In summary, current evidence positions APNs as key to operationalizing the principles of the CCM and advancing WHO’s vision for high-performing primary care. Yet, while outcomes are well documented, *the processes through which APNs generate value*, particularly in terms of communication, interprofessional collaboration, and integration of care, remain insufficiently understood.

## Research design and methods

### Theoretical framework: the chronic care model

CCM was developed to improve the management of chronic illness by promoting proactive, patient-centered, and coordinated care [25]. It comprises six interrelated components: Self-management support, delivery system design, decision support, clinical information systems, healthcare organization, and community resources. Together, these elements emphasize a systemic approach that goes beyond individual encounters and highlights the interdependence between patients, professionals, organizations, and communities.

As WHO [26] underlines, continuity and coordination of care are essential to achieving people-centered health services, particularly for patients with multimorbidity. The CCM thus offers a useful analytical lens for exploring how both structural and relational dimensions of care contribute to coherence and quality in community-based healthcare [27].

However, while the CCM emphasizes productive interactions between informed patients and proactive care teams, existing research provides limited insights into *how* such interactions are achieved in everyday practice. In particular, the role of APNs in facilitating communication, coordination, and collective sensemaking across sectors remains underexplored.

In this study, the CCM is applied and extended to investigate *how APNs create value* for patients, relatives, and professional collaborators by operationalizing continuity of care, coordination, and self-management support in complex care trajectories. This framework guides the analysis by linking APNs’ communicative and integrative practices to the systemic mechanisms that underpin people-centered, community-based care.

### Research design. A qualitative case study

This study employed a qualitative case study design [28], with Aalborg Municipality (serving approximately 220,000 inhabitants) as the empirical case to explore how APNs operate in and bring value to an established primary care setting. The case was selected due to its uniqueness in a Danish context and its mature implementation of the APN role, embedded in cross-sectoral collaboration between general practice and municipal healthcare services.

A multi-method strategy was applied to capture complementary perspectives and to strengthen analytical validity through empirical triangulation [29]. By combining interviews with patients, relatives and external stakeholders and a reflective workshop with APNs, this study illuminated both external experiences and internal professional reasoning. This approach enables exploring not only what works, but also for whom, how, and why, thereby opening the “black box” of complex care trajectories [30–32].

#### Case description and local APN model

**Fig. 1:**
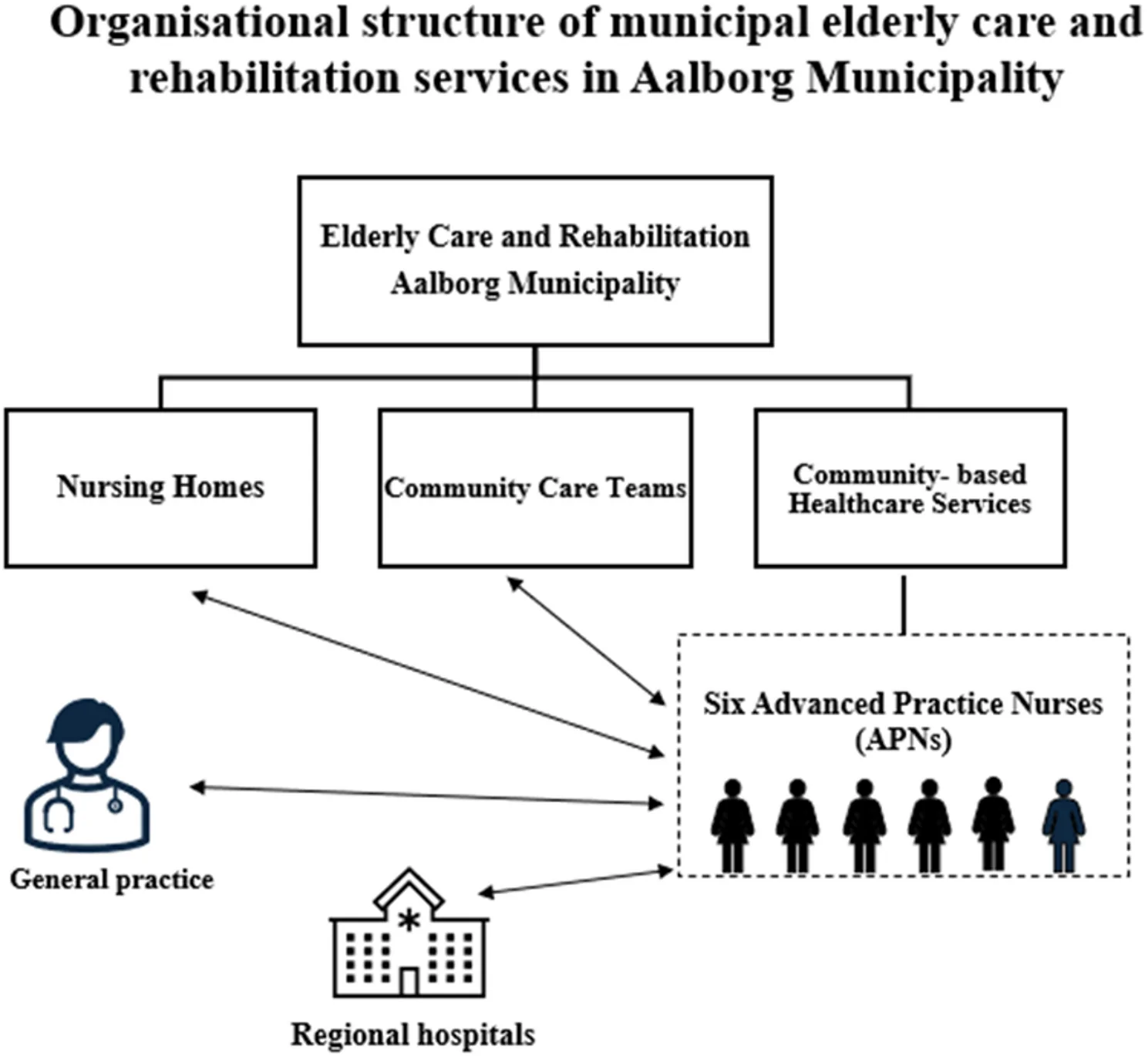
Organisational anchoring of the Advanced Practice Nurse (APN) function within municipal health, rehabilitation, and elderly care services in Aalborg Municipality

This study was conducted as a qualitative case study of a municipally implemented APN function in Aalborg Municipality, Denmark. The case comprised community-based healthcare services involved in the care of adults with multimorbidity and complex care needs, including home care units, nursing homes, a short-stay facility, and collaborating general practices within the municipality. As illustrated in Fig. 1, the case boundaries are defined by the organisational and professional interfaces through which APNs operate across municipal services and collaborate with general practice and hospital care.

In Aalborg Municipality, APNs are registered nurses with a master’s level education in advanced practice nursing and are positioned to work across settings rather than within a single organisational unit. Their role includes advanced clinical assessment, coordination of complex care trajectories, and close collaboration with general practitioners, municipal care professionals, and patients and relatives. While APNs work with a high degree of professional autonomy within the municipal organisation, decisions regarding medical treatment are made in collaboration with general practitioners.

This locally implemented APN model constitutes the empirical case examined in the present study and provides the contextual basis for interpreting the findings and their potential transferability.

#### Case

The primary healthcare sector is the frontline of the Danish healthcare system, which is largely tax-funded and free of charge. The primary healthcare sector comprises general practitioners (GPs), municipal health services such as home care, nursing and rehabilitation, health visitors, and privately practicing specialists. GPs function as gatekeepers to specialist and hospital care, while municipalities are responsible for community nursing, nursing homes, long-term care, and health promotion. The primary healthcare sector is thus central to managing patients with chronic conditions and multimorbidity as well as coordination across care settings.

In this study, the term *patient* refers to citizens receiving municipal healthcare services, including home nursing, rehabilitation, and nursing home care. The term *patient* is used throughout the article to ensure clarity for an international readership, although municipal healthcare users are often referred to as *citizens* in the Danish context.

#### Context of the APN function in Aalborg Municipality

Aalborg Municipality is the third largest in Denmark, with a diverse population and a growing number of elderly. The municipality has a well-developed primary health care infrastructure, including municipal nursing services, nursing homes, rehabilitation units and close collaboration with GPs.

#### The APN function in Aalborg Municipality

Since 2021, Aalborg Municipality has implemented a structured APN model in the municipal health system. In 2024, six APNs had been employed and strategically positioned to work across nursing homes and home care services in close collaboration with GPs and hospitals. In this capacity, the APN function operates both inter-professionally and across sectors aiming at ensuring continuity, coherence, and high-quality care.

#### Target group and scope of the APN function

The APN function in Aalborg Municipality is directed at the 2–5% of patients living with complex health conditions, frailty, and/or multimorbidity. Interventions take two main forms: (1) APN pathways offering advanced nursing in complex patient cases, and (2) APN consultation providing advice and support to other professionals.

Referral into the APN function was guided by a locally developed *APN triage* framework, which systematically assesses the medical, social, and organizational complexity to identify patients who would benefit from APN-led care [33, 34].

APN triage provides a structured, holistic approach to identifying and supporting patients with complex health needs. It operates at both individual and organizational levels and should be regarded as a dynamic tool, continuously adapted in line with ongoing quality development.

#### Characteristics of patients in APN pathways

As part of the municipal implementation and ongoing development of the APN function, routinely compiled documentation has been used to describe the overall characteristics of patients enrolled in APN-supported care trajectories. To date, aggregated data from more than 150 cases have been compiled for service development and quality purposes.

This aggregated descriptive data was used in the present study solely to provide contextual background for the study setting. These data were not collected specifically for research purposes and were accessed and reported only in anonymised and aggregated form. They were not subjected to analytical procedures and were not linked to individual participants.

The contextual data include demographic variables (age, gender, marital status), selected chronic conditions, medication use, hospital admissions within the past year, and frailty assessed using the Clinical Frailty Scale (CFS). Additional descriptors relate to housing conditions, extent of health and social services received, social support, and the number and type of professionals involved in each case.

Overall, the aggregated data indicate that patients referred to APN pathways span all adult age groups and have an approximately even gender distribution. A majority were single, and most lived in their own homes. The data further suggest a high prevalence of multimorbidity, including cardiovascular disease, diabetes, chronic obstructive pulmonary disease, and cognitive impairment.

Polypharmacy was common, with most patients receiving more than five daily medications. Approximately one third were living with mental illness or depression, and a similar proportion had current or previous substance use problems. The data also reflect high levels of healthcare utilisation, including frequent hospital admissions and involvement of multiple providers across health and social care.

Frailty scores documented in routine municipal records indicate that many patients lived with mild to moderate frailty (mean CFS 5.7), corresponding to a need for support with daily activities such as cooking, housekeeping, and personal hygiene.

#### Two main categories of patients within the APN target group

Based on aggregated descriptive data documented in routine municipal records, two main categories of patients are commonly observed within the target group for APN-led care. The first category consisted of older (around 80 years), multimorbid and frail patients characterized by frequent hospital readmissions, polypharmacy, and high levels of comorbidity. The second category comprised somewhat younger patients, spanning from their early twenties to older adults, who have both somatic and psychiatric conditions, often in combination with substance abuse, reduced health literacy, and a limited social network. Across both groups, approximately one fifth were described as particularly challenging, as they resist collaboration and actively decline support from the healthcare system, including APN-led services.

### Data collection strategy and empirical material

#### Sampling and recruitment

Participants were purposively recruited to ensure variation in perspectives across patient, relative, municipal, and general practice contexts. Patients and relatives were identified through the APN function, while municipal care professionals and general practitioners were recruited based on their direct collaboration with APNs. Efforts were made to include participants with diverse experiences of APN involvement, including both well-functioning and challenging care trajectories.

#### Data collection phases

To achieve a comprehensive and nuanced understanding of how APNs create value in municipal healthcare, data collection was structured in two complementary phases. Phase one gathered experiences and perspectives from patients, relatives, municipal collaborators, and general practitioners through individual and group interviews, thereby documenting external viewpoints on APN practice. Phase two consisted of a reflective workshop with the APNs, designed both to validate and expand the interview findings and to articulate dimensions of APN practice that are often tacit or invisible to external stakeholders. These two phases together produced a robust empirical foundation integrating external perspectives with APNs’ own professional reasoning, which enabled a deeper analysis of the APN role and practice.

#### Researcher reflexivity

The first author conducted all interviews and has a background in nursing and qualitative health research. This professional familiarity with the field enabled in-depth exploration of practice but also required ongoing reflexive awareness. Reflexivity was addressed through continuous analytic dialogue between the authors, critical questioning of interpretations, and explicit attention to alternative explanations during analysis.

#### Phase 1: Interviews

In the first phase, qualitative data were collected through individual and group interviews with 24 informants purposively recruited for their direct involvement in, or experience with, APN-led support in Aalborg Municipality. Five patients and two relatives participated in in-depth individual interviews, while eleven municipal care professionals (including nurses and social and healthcare assistants) were interviewed in five focus groups. In addition, five GPs and one general practice nurse took part in individual semi-structured interviews. Interviews lasted between 30 and 90 min, were conducted either in person or via secure digital platforms, audio-recorded, and transcribed verbatim. All interviews followed semi-structured guides developed for each informant group. These guides, along with written participant information and consent materials, are provided in *Additional file 1: Interview and workshop guides*.

All interviews were conducted in Danish. Quotations included in the manuscript were translated into English by the first author. The translated quotations were reviewed by a bilingual co-author, and final wording was agreed by consensus.

**Table 1:** Overview of interview informants and empirical material (Phase 1)

Informant group	Number of informants	Interview type and format	Data collection period	Volume of material
Patients	5	5 in-depth, semi-structured indivi-dual interviews	May–June 2025	4 h 10 min, 92 pages
Relatives	2	2 in-depth, semi-structured indivi-dual interviews	May–June 2025	2 h 40 min, 62 pages
Municipal care professionals	11 [6 specialized nurses, 4 registered nurses, 1 social and healthcare assistant]	5 focus group interviews	June–July 2025	2 h 50 min, 124 pages
General Practitioners [GPs] and general practice nurse	6 [5 GPs, 1 general practice nurse]	6 individual, semi-structured interviews	July–August 2025	5 h, 128 pages
Total	24 informants	18 interviews (13 individual, 5 focus groups)	May–August 2025	14 h 40 min, 406 pages

Table 1 provides an overview of the empirical material, including informant groups, interview formats, data collection periods, and the total volume of transcribed data.

#### Phase 2: Reflective workshop with APNs

In the second phase, a reflective workshop was conducted with the six APNs in Aalborg Municipality and their leader (*n* = 7) (see Table 2). The purpose was to present and discuss key findings from the interviews conducted in Phase 1, thereby creating a space for reflection, validation, and further elaboration of empirical insights. This methodological step was informed by principles of triangulation and member checking [35], as it incorporated APNs’ own perspectives to nuance and qualify themes identified across the other informant groups. Materials used for the reflective workshop, including the semi-structured guide and written information provided prior to participation, are included in Additional file 1.

The workshop was organized as a semi-structured reflective session, in which selected quotes and themes from the previous analyses were used as prompts for joint reflection. The workshop was audio-recorded, transcribed verbatim, and coded exploratively in line with the rest of the data material. This process allowed for articulation of dimensions of APN practice that are often less visible to patients and collaborators, such as clinical reasoning, coordination tasks, and relational micro-practices.

Thus, the workshop functioned both as a validation of the identified findings and as supplementary empirical material, capturing APNs’ own understanding of their role and value creation in primary healthcare.

**Table 2:** Overview of informants and empirical material (Phase 2)

Informant group	Number of informants	Interview type and format	Data collection period	Volume of material
APN nurses and their leader	6 APNs, 1 leader (*n* = 7)	Semi-structured reflective workshop	September 2025	1 h 30 min, 18 pages

### Analytical strategy

All qualitative data were thematically analyzed using Braun and Clarke’s six-phase approach [36]. This was followed by a theoretically informed interpretation guided by the CCM. To ensure consistency and transparency, the analysis was conducted jointly by the two authors.

Four dimensions of the CCM were identified as particularly relevant for understanding APN practice in Aalborg Municipality: (1) Proactive organization and clear role distribution (delivery system design), (2) Integration of evidence-based knowledge and clinical reasoning (decision support), (3) Empowerment of patients and relatives (self-management support), and (4) Mobilization of broader social and community resources (community resources). Consistent with WHO’s emphasis on continuity in and coordination of care as core features of people-centered health systems [26], these dimensions formed the theoretical framework of the analysis.

### Findings

#### Analytic approach and development of themes

The analysis was conducted inductively across the full dataset, including interviews with patients, relatives, municipal care professionals, general practitioners, and the reflective workshop with APNs. Initial coding focused on identifying recurrent patterns related to APN involvement in complex care trajectories. Through iterative comparison across informant groups, codes were clustered into subthemes that captured shared processes and practices rather than stakeholder-specific perspectives.

Two cross-cutting themes were subsequently developed, reflecting mechanisms that recurred across settings and informant groups. These themes were not predefined but emerged inductively from the data. In a second analytic step, the themes were interpreted in relation to the Chronic Care Model (CCM) to examine how APN practices contributed to integrated and person-centred care. This theoretically informed interpretation was used to situate the empirically derived themes within an established framework, while maintaining analytic openness to tensions, boundary issues, and less positive accounts present in the data.

The two themes thus represent process-oriented findings that cut across professional and organisational boundaries and illuminate how APNs enact integration in everyday primary and community-based care.

The findings are presented in relation to the dimensions of the CCM. Thematic analysis across all informant groups revealed recurring patterns that not only align with but also extend the CCM by highlighting the communicative and integrative mechanisms through which APNs generate value in complex care. Two cross-cutting themes emerged as central: (1) APNs as strategic communicators and accountable coordinators, and (2) APNs integration of competences from fundamental to advanced nursing.

The section first presents the value of APNs as experienced by key stakeholder groups, followed by an in-depth analysis of the two cross-cutting themes and their relation to existing theoretical frameworks.

### Value of APNs for key stakeholders

#### Value of APNs for patients and relatives

For patients and relatives, the value of APNs was experienced as deeply relational and tangible in everyday life. A recurring theme was relational security and continuity, grounded in the APNs’ ability to build trust, listen actively, and remain present in encounters. Patients consistently described APNs as *kind*,* professional*,* and trustworthy*, emphasizing that the relational work was not an optional supplement but a prerequisite for feeling safe and acknowledged in fragmented trajectories. As one patient expressed: “*She had time – I didn’t feel like just another number in line*.” Similarly, relatives highlighted the relief of being seen and supported alongside their loved one: “*As a relative*,* it means so much that someone also sees me and my father as a person*,* and takes the time to understand his situation*,* which is so difficult and overwhelming*.”

A second dimension of value lies in assumption of responsibility and coordination across complex care trajectories. Patients and relatives described APNs as key figures who “tie the threads together” across general practice, community-based municipal nursing, hospitals, and social services, thereby alleviating the heavy burden of navigating between multiple providers. As one daughter noted: “*It is such a relief that it’s not only my responsibility to get everything in place for my father – I was close to breaking down.*”

Third, clinical competences and advanced skills of APNs were perceived as inseparable from their relational approach. Medication reviews, systematic assessments, and thorough follow-up are directly linked by patients to improve health and quality of life. One patient explained: “*When she went through my medication*,* a whole series of pills disappeared – I think I actually got sicker from some of them. Now I feel much better*.” Such interventions were described as life-changing in terms of coping, stability, and the capacity to live more independently.

Finally, patients and relatives emphasized the role of APNs in health communication – “translating” medical language and treatment plans into understandable terms and making sense of complex systems. This communicative function strengthened both confidence and self-efficacy: “*It is nice that she can explain things*,* so I understand them – she was really good at that*.”

Patients and relatives experienced APNs as creating value through a combination of relational presence, accountable coordination, advanced clinical competences, and accessible communication. This integrated contribution improved not only health outcomes but also everyday sense of security, coping, and the feeling of not being left alone in a fragmented health system.

#### Tensions and limits of perceived value

While APN involvement was generally described as adding value to complex care trajectories, the analysis also identified situations in which APN-led care did not result in perceived benefit or was actively declined by patients. These cases illustrate important limits to APN value creation and highlight the centrality of patient preferences in complex trajectories.

One APN described a patient with multiple chronic conditions who did not perceive a need for APN involvement and explicitly declined clinical assessment and coordination support. As the APN explained: “*I have a patient right now who does not see the need for me. She does not want me to carry out clinical assessments… the only thing I have really been able to do is help her apply for a grant*.”

Despite the patient’s clinical complexity, APN engagement was constrained by the patient’s own understanding of her situation and her preference to rely on informal support rather than professional coordination: “*She has many illnesses*,* but she does not see it as a problem and would prefer her friends to help*.”

From an APN perspective, this created a tension between professional assessments of complexity and respect for patient autonomy. Although other professionals had identified the patient as potentially benefiting from a coordinating role, the APN’s contribution remained limited: “*There are others who have thought that she could benefit from having a care coordinator. That perspective is something I would like to make visible*.”

Analytically, this case demonstrates that APN involvement does not automatically translate into perceived value for patients, even in highly complex situations. Rather, APN contributions depend on patients’ willingness to engage and on alignment between professional assessments and patients’ own priorities. These findings nuance assumptions about APNs’ coordinating role and underscore that integration and continuity cannot be imposed but must be negotiated within each care trajectory.

In addition to patient-related constraints, the analysis also highlighted organisational and structural limitations to APN value creation. APNs described how organisational, legal, and administrative rigidities within and across sectors at times restricted their ability to act on identified needs, particularly for patients with complex social and mental health challenges.

In these situations, APNs could identify unmet needs and potential interventions, but lacked mandate or available pathways to translate assessments into concrete action. As a result, APN involvement was experienced as limited not by clinical complexity, but by structural barriers that constrained coordination, follow-up, and continuity across services.

#### Value of APNs for municipal collaborators

Across five focus groups with nurses and a social and health care assistant from four community-based nursing units and one short-stay facility, APNs were consistently described as catalysts for professional learning and quality development. Collaborators highlighted how APNs introduced specialized knowledge, asked reflective questions, and opened new avenues for problem-solving, thereby creating durable learning spaces in everyday practice: “*They ask reflective questions*,* create professional curiosity*,* and open new doors for possible understandings and solutions*.” The presence of APNs in conferences and case meetings was said to “*raise the level of practice*” for both specialized and general nurses and to enable progress in patients with very low health literacy.

A second prominent theme was the holistic, analytical, and responsibility-taking approach of APNs in highly complex cases. Collaborators emphasized that APNs combine advanced clinical assessment with a system-level perspective, detect underlying drivers of deterioration, and intervene early often preventing avoidable admissions and breaking negative spirals. Crucially, APNs assume ownership of “orphaned” tasks with no clear “systemic ownership”: “*They take responsibility for tasks that no one else clearly owns - which is a system problem*.” Their longitudinal chart reviews and synthesis of loose ends were considered pivotal: “*She dug far back in the record and found connections and unresolved problems behind the repeated admissions*.”

Third, APNs functioned as bridge-builders and collaboration catalysts when complexity exceeds the capacity of frontline teams: “*When trajectories become so complex that our competences no longer suffice*,* we call them in*.” By convening the right stakeholders and ordering interventions, they align efforts so that “*what each of us is best at is unfolded in the right sequence*.” Collaborators also stressed APNs’ respectful and professional style, which sustains a psychologically safe learning climate: “*They’ve never made me feel stupid when I asked for help - that’s essential for being open about doubts*.”

In sum, municipal collaborators experienced APNs as advanced clinicians who also filled a systemic coordination gap. They contributed to reflection and learning in everyday practice, improving skills and assuming accountable ownership of unclaimed work. At the same time, they orchestrated interprofessional interventions, action in the right order - thereby stabilizing chaotic trajectories and lifting the overall quality of care.

#### Value of APNs for general practitioners and practice staff

For GPs and general practice staff, APNs were experienced as a unique resource in the handling of complex patient trajectories. A central theme was their holistic and integrative perspective, where medical, psychiatric, and social dimensions were combined into a coherent frame. Several GPs described how APNs assumed responsibility for tasks that traditionally fall within the medical domain, such as medication reviews and dialogue with hospitals about treatment decisions. One GP reflected, *“In this case*,* the APNs were in contact with the hospital about whether a pleural drain should be removed – a task I would normally be expected to handle*,* but which they competently managed.”*

Collaboration was consistently described as grounded in trust, respect, and continuity, often evolving into genuine trust-based partnerships. These partnerships generated renewed professional energy in cases otherwise close to collapse, *“I had a partnership with this APN which was so valuable to me. It gave me energy in a case I had practically given up on – and it also gave the patient new energy.”* Such experiences highlight that APNs not only provide clinical relief but also enable doctors to persist with demanding cases.

At the same time, APNs’ respectful and loyal approach ensured that task-sharing did not undermine professional roles. GPs emphasized that although role boundaries sometimes overlap, APNs’ sensitivity and loyalty prevented tension: *“It’s great when the APN sees the need for a medication review – but it must be done with care and loyalty*,* so as not to expose the GP’s shortcomings. And I’ve never experienced them doing otherwise.”* This approach fostered an atmosphere of equality and constructive dialogue, strengthening both collaboration and patient safety.

Finally, GPs pointed to the ability of APNs to act as key persons in navigating the complexity of primary care. Their presence was considered indispensable in cases involving multimorbidity, psychiatric comorbidity, or social vulnerability, where the limits of general practice became apparent. As one GP noted: *“We can’t do much alone – especially not for this complex group. There is a great need for nursing competences that encompass psychiatric and social dimensions.”* By anchoring their interventions in patients lived realities, APNs expanded the scope of primary care and provided a depth that many GPs found difficult to achieve within existing system constraints.

Thus, GPs and practice staff experienced APNs as trustworthy partners who combine holistic clinical competences with relational sensitivity. They bring relief in a pressured everyday practice, create energy and persistence in highly complex cases, and foster genuine partnerships built on respect and shared responsibility.

### APNs as strategic communicators and coordinators in interprofessional collaboration

Across the empirical material, APNs emerged as pivotal not only for coordinating collaboration but also for strategically shaping the communicative processes that sustain it. Rather than merely adding clinical capacity, they acted as accountable coordinators who gather stakeholders, facilitate dialogue, and assume responsibility for coherence in complex care trajectories. As one APN put it:

"*You can be the most skilled clinical expert, but if you are not able to collaborate or establish collaboration, you will not succeed*".

This perspective underscores that collaborative competence is not an optional supplement but a fundamental precondition for the ability of APNs to apply their advanced expertise and ensure sustainable partnerships across professional and organizational boundaries.

As another APN reflected during the workshop:

*“It is us. We also carry the responsibility of bringing partners together*,* calling them up*,* and acting as the patient’s advocate. For those patients who have not had a voice in the various collaborations – whether with the GP or the hospital – it is us who establish the contact and make sure communication flows between partners. Our agenda is to create coherence*,* which means we also have the responsibility to secure the best possible collaboration”.*

Municipal collaborators also highlighted the systemic role of APNs:

"*They take responsibility for tasks that no one else has clear ownership of – which is a systemic problem*".

GPs described collaboration with APNs as developing into genuine partnerships, characterized by mutual responsibility and renewed professional energy:

"*I had a partnership with this APN, which was so valuable to me. It gave me energy in a case I had practically given up on – and it also gave the patient new energy".*.

Importantly, both nurses in primary care and GPs emphasized that APNs established collaboration without undermining colleagues or exposing professional shortcomings. Instead, their approach was experienced as respectful, constructive, and conducive to learning. As one community-based nurse noted, *“They never make you feel inadequate when you ask for help – and that is essential for being open about doubts and uncertainties.”* Likewise, GPs emphasized that APNs’ clinical suggestions were constructive, enhancing shared decision-making rather than criticism of medical practice.

In summary, the empirical material showed that APNs’ collaborative competences are rooted in their ability to flexibly navigate communicative strategies and facilitate collective sensemaking. By moving between informing, negotiating, and co-owning decisions, they foster mutual learning, patient-centered goal-setting, and sustainable partnerships across professional and organizational boundaries. This positions APNs not only as clinical experts, but also as key communicative stakeholders who extend the CCM’s notion of delivery system design into the everyday relational practices that underpin integrated care [37].

### From basic to advanced nursing

A central theme across the empirical material was the ability of APNs to move seamlessly between basic and advanced nursing practices. APNs emphasized that advanced practice cannot be enacted without simultaneously mastering and applying fundamentals of care.

A distinctive example of how APNs integrate fundamental and advanced nursing in practice is the *APN triage*, a locally developed framework used to identify and manage complexity (Table 3). The APN triage is a locally developed practice tool implemented in Aalborg Municipality and has not been formally validated beyond this context. The framework has been iteratively refined through ongoing clinical use as part of local quality development.

APN triage is a holistic assessment tool designed to identify and prioritize patients with complex health needs. Drawing on empirical data, clinical experience, and relevant literature, the triage evaluate each citizen across six parameters: Frailty (assessed with the Clinical Frailty Score), symptom burden, health literacy, social resources, hospital admissions, and number of professional stakeholders involved. Inspired by Vinge’s typology of medical, social, and organizational complexity [34], the APN triage translates these dimensions into practice-oriented concepts that guide assessment and prioritization. Details of the APN triage domains and indicators are presented in Table 3.

**Table 3:** The APN Triage Framework: Domains and Indicators of Complexity. Adapted from the APN Triage developed by APN in Aalborg Municipality

Domain	Focus	Illustrative indicators / examples
**Medical** **complexity**	Clinical frailty and cumulative symptom burden affecting daily functioning and quality of life [33, 38].	CFS ≥ 5; multiple chronic conditions; high symptom burden.
**Social** **complexity**	Health literacy and strength of social networks influencing coping, self-management, and engagement with services [39, 40].	Limited health literacy; social isolation; unstable housing or support.
**Organizational complexity**	Degree of fragmentation and number of providers across sectors, indicating coordination needs [34].	≥ 3 hospital admissions per year; > 5 professionals involved; multiple interfaces.

The APN triage enables nurses to translate multidimensional complexity into assessable and actionable insights, guiding prioritization, coordination, and relational care across sectors. It exemplifies how advanced practice can generate context-specific frameworks that operationalize WHO’s call for structured, people-centered coordination in primary healthcare.

This integrative approach was also recognized by patients, relatives, and professional collaborators as a defining feature of APNs. Through the framework, APNs combine advanced clinical reasoning with holistic and relational awareness. They identify not only medical instability but also social and organizational barriers that often drive complexity - such as low health literacy, lack of support networks, or fragmented service interfaces. The triage thereby exemplifies how APNs operationalize advanced practice by integrating assessment, coordination, and relational care in a coherent, patient-near process.

As one APN explained during the reflective workshop:

*“Well*,* this is basic nursing. It’s the fundamentals of care – simply the basics… And in order to deliver advanced nursing*,* you also need to master and deliver the basic nursing”.*

Because APNs in this context work directly with patients in their homes and in close clinical practice, they are uniquely positioned to mobilize their full spectrum of competences. This allows them to address basic care needs, psychosocial barriers, and advanced clinical problems within the same encounter.

Patients, municipal collaborators, and GPs consistently emphasized that APNs anchor their interventions in patients’ own perceived problems - questions that are often not asked elsewhere. APNs themselves described that by approaching encounters openly and systematically mapping all issues, including those prioritized by patients, they were able to detect the fundamental needs that frequently turn out to be the most complex challenges in cases of multimorbidity and hypercomplex trajectories.

One example illustrates how fundamental and advanced levels intertwine:

"*He couldn’t eat because his dentures were too big, and he had no IT skills to complete the application needed for new ones. So, he couldn’t get replacements, which meant he couldn’t eat, and this led to undernutrition, dehydration, and repeated hospitalizations. The root problem was not medical per se, but the lack of IT competences".*.

For patients and relatives, this integration of the fundamental and the advanced was experienced as a holistic approach, where relational presence and clinical thoroughness are inseparable:

*“She (the APN nurse) asked me what my biggest problem was… and it wasn’t my diseases*,* but that I had a bad life that I wasn’t in control of. She met me with respect and gave me back a sense of dignity”* (Patient).

*“She [the APN nurse] really went thoroughly through everything – I think she was here for a couple of hours and asked about everything from head to toe.”* (Patient).

These experiences reflect how relational respect and recognition are integral to APNs’ capacity to restore patients’ sense of dignity and agency in complex trajectories.

GPs also highlighted the ability of APNs to combine medical, psychiatric, and social perspectives in a way that bridges disciplinary silos:

*"She could talk to the patient the way we do in practice: about skin, incontinence, psychiatry, and practical issues all together – in a very elegant and competent way".*

Taken together, these perspectives illustrate how APNs’ integration of relational, clinical, and organizational competences creates coherence across professional boundaries and restores continuity for patients with complex care needs.

## Discussion

### Contribution to existing knowledge and theoretical implications

This study advances theory by extending the CCM with relational and communicative dimensions that explicate how integration and continuity are enacted in everyday practice. It addresses a re-cognized international knowledge gap [20] by illuminating the *mechanisms* through which APNs generate value in community-based care.

Whereas most existing studies document *what* APNs achieve in terms of outcomes, this study demonstrated *how* APNs achieved impact by integrating fundamental nursing care with advanced clinical reasoning, system navigation, and communicative coordination in patient-near contexts.

### Integration of fundamental and advanced practice

These findings nuance the CCM’s dimensions of *decision support* and *self-management support*. The APNs provided structured clinical guidance but simultaneously addressed practical, social, and existential barriers that determine whether patients can implement treatment plans and sustain self-care. Taking the starting point in patients perceived problems and mapping the full complexity of their situation, APNs operationalized the CCM’s call for *productive patient–provider interactions*, ensuring that medical advice is anchored in the lived realities of multimorbid and hypercomplex patients.

The findings also substantiate the Fundamentals of Care (FoC) framework [41, 42], which conceptualizes high-quality nursing as the integration of: (1) The nurse–patient relationship, (2) Physical, psychosocial, and relational needs, and (3) The care context. APNs exemplify this integration by combining relational engagement (what does the patient think is the biggest problem? ), clinical thoroughness (systematic assessments, medication reviews), and contextual problem-solving (e.g., IT barriers, coordination with social services).

By demonstrating how APNs mobilize all their competences from basic care to complex clinical reasoning within the same encounter, the study provides a process-oriented contribution to international evidence. It shows that *the fundamental often becomes complex* and that sustainable outcomes depend on the integration of relational, organizational, and clinical domains of care. In doing so, the findings extend the CCM’s understanding of self-management support and decision support, showing that these dimensions are not confined to guidelines or patient education but also include the interplay of fundamental care, advanced reasoning, and lived experiences as the foundation for continuity and quality.

### Communicative and collaborative mechanisms

Beyond clinical integration, this study contributes new insights into APNs’ communicative and relational mechanisms. Global reviews highlight persistent gaps regarding how APNs contribute to interprofessional collaboration and team functioning [20].

These findings align with theoretical perspectives on interprofessional communication [37], which conceptualize collaboration as a continuum of communicative strategies ranging from informing and exchanging information to negotiating meaning, agreeing on goals, and ultimately sharing responsibility for decisions and joint action. The empirical material illustrated how APNs moved flexibly along this continuum: informing and updating GPs through written correspondence, exchanging observations with municipal colleagues, negotiating and aligning goals with patients and relatives, and - in the most complex cases - co-creating decisions and sharing responsibility across professional boundaries.

As Fox, McAllum & Mikkola [37] further argue, the more complex a patient or care trajectory becomes, the more integrated and mutually dependent collaborations need to be, and the greater the need for collective sensemaking. APNs were both recognized by collaborators and saw themselves as responsible for facilitating this sensemaking - piecing together fragmented information, aligning perspectives, and ensuring that shared understanding translated into coordinated action.

In this way, APNs demonstrated advanced collaborative competence: they not only contributed clinical expertise but also actively managed the communicative dynamics through which interprofessional collaboration became productive. By balancing respectful partnership with strategic navigation of hierarchy, they stabilized fragile professional relations while enabling collective problem-solving.

Importantly, these communicative strategies were enacted with respect for professional boundaries and in ways that fostered trust, mutual learning, and shared ownership of care. Both GPs and community-based nurses described APNs’ collaborative style as constructive and inclusive, enabling open reflection and joint problem-solving rather than reinforcing hierarchy.

In complex trajectories, APNs thus managed the communicative infrastructure that makes integrated healthcare work. Their role extends the CCM’s notion of delivery system design into the everyday micro-practices that sustain cross-sectoral collaboration in primary care.

### Practice innovation and policy relevance

The APN triage developed in Aalborg Municipality exemplifies how advanced practice can produce context-specific frameworks that make complexity *assessable and actionable*, thereby operationalizing WHO’s call for structured, people-centered coordination in primary care [3, 26]. It illustrates how local practice innovation can serve as a transferable model for implementing global policy goals of integration and equity.

Together, these mechanisms position APNs as *boundary-spanning professionals* who transform care coordination and system integration from abstract policy ambitions into concrete, everyday clinical reality. Their contribution lies not only in what they do, but in *how* they weave together the communicative, relational, and clinical threads that ensure coherent and person-centered care.

### Strengths and limitations

This study has several strengths. The multi-perspective design, encompassing patients, relatives, municipal care professionals, general practitioners, and APNs, provides a comprehensive and nuanced understanding of how APNs create value in complex community-based care. The focus on process-oriented mechanisms and a mature, real-world implementation enhances the analytic depth and relevance of the findings for primary care organisation and professional practice.

Some limitations should be acknowledged. As a single-case study conducted in one Danish municipality, the findings are context-specific and analytically rather than statistically transferable. Moreover, participants were recruited through existing APN pathways, which may entail a risk of positive selection. However, the inclusion of multiple informant groups and empirically grounded disconfirming cases strengthens the credibility of the analysis and supports cautious transferability to comparable primary and community-based care settings.

## Conclusion

Taken together, the findings demonstrate how Advanced Practice Nurses create value in community-based care for people with highly complex needs through relational, communicative, and coordinative practices embedded in everyday primary care.

In doing so, the study contributes process-oriented knowledge on how APNs support integrated and coherent care trajectories, not primarily through isolated clinical interventions but through everyday practices of coordination, communication, and relational work.

By integrating fundamental nursing care with advanced clinical reasoning and system navigation within patient-near encounters, APNs are positioned to address complexity as it unfolds across sectors and professional boundaries. Their role highlights how continuity and integration in primary care depend on the alignment of clinical, relational, and organizational domains of practice.

At the same time, the findings indicate that APN contributions are not unconditional but depend on patient acceptance and on organisational and governance structures that enable coordinated action across sectors.

These insights advance understanding of advanced practice nursing by explicating how impact is generated in complex care trajectories. Future research should examine how such processual mechanisms operate across different organizational contexts and populations to inform the further development of APN roles in primary care.

### Implications for practice and policy

The findings suggest that Advanced Practice Nurses can support integrated care in primary and community-based settings by facilitating coordination across sectors and aligning clinical, relational, and organizational aspects of care for people with multimorbidity. Rather than substituting existing professional roles, APNs appear to strengthen care trajectories by integrating fundamental nursing care, advanced clinical reasoning, and close patient engagement within everyday practice.

From a policy perspective, the study indicates that realizing the potential of advanced nursing roles in primary care requires organizational and governance frameworks that support professional autonomy, cross-sectoral collaboration, and advanced clinical training. Locally developed practice models, such as the APN triage described in this study, may offer context-sensitive examples of how broader policy ambitions for integrated, people-centred primary healthcare can be operationalized in practice.

## Future directions for research and evaluation

This study provides in-depth qualitative insights into how APNs create value through communicative strategies, competence integration, and cross-sectoral collaboration in complex, community-based care. By incorporating perspectives from patients, relatives, municipal healthcare professionals, GPs, and APNs themselves, the study contributes with a rare process-oriented understanding of the mechanisms and contextual conditions that enable APN-led care to succeed.

As a qualitative case study anchored in a single municipality, the findings are not intended to be statistically generalizable. Instead, they offer analytical generalizability by illuminating mechanisms that can inform both practice and policy across contexts. Nevertheless, further research is needed to complement these insights with broader evidence. Quantitative and longitudinal evaluations could strengthen the documentation of APN-led care in terms of outcomes, efficiency, and cost-effectiveness. Comparative studies across municipalities and countries could reveal contextual enablers and barriers, while patient-reported outcomes and experiences (PROMs and PREMs) would enrich understanding of APNs’ impact on quality of life, self-management, and equity—particularly among vulnerable groups.

Finally, mixed methods approaches that integrate outcome data with qualitative accounts of collaboration, communication, and competence integration would bridge the gap between *what works* and *how it works*. In this way, future research can build on process-oriented knowledge and expand the international evidence base on APNs’ role in strengthening integrated, people-centered primary healthcare.

## Perspectives

The findings of this study resonate strongly with the broader policy agenda articulated by WHO, which calls for strengthening primary and community-based care as a response to rising multimorbidity, hospital overcrowding, and the need for sustainable, people-centered health systems [20, 26]. In this context, APNs illustrate how such a shift can be operationalized in practice. By integrating fundamental and advanced nursing, and by strategically managing interprofessional communication and collaboration, APNs contribute to care pathways that are both clinically sound and relationally coherent.

From a system perspective, this positions APNs not as supplementary staff but as strategic enablers of WHO’s vision for integrated, equitable, and sustainable primary healthcare. Their dual capacity to address the fundamentals and the complex simultaneously, and to facilitate collective sensemaking across sectors demonstrates how professional roles can be reconfigured to meet demographic and organizational challenges. Scaling up APN roles therefore requires investment not only in advanced education and professional autonomy but also in organizational structures that recognize and leverage their process-oriented value.

In short, APNs exemplify the type of embedded, relationally competent and clinically advanced professionals that are needed to fulfil WHO’s call for high-performing primary healthcare systems capable of addressing complexity while promoting equity and continuity.

## Supplementary Information

Supplementary Material 1

## Data Availability

The datasets generated and analyzed during the current study are not publicly available due to privacy restrictions but are available from the corresponding author on reasonable request.

## Abbreviations

APN: Advanced Practice Nurse
CCM: Chronic Care Model
CFS: Clinical Frailty Scale
CNS: Clinical Nurse Specialist
FoC: Fundamentals of Care
GP: General Practitioner
NPs: Nurse Practitioners
WHO: World Health Organization

## References

[1] Barnett K, Mercer SW, Norbury M, Watt G, Wyke S, Guthrie B. Epidemiology of Multimorbidity and implications for healthcare, research, and medical education: a cross-sectional study. Lancet. 2012;380(9836):37–43. 10.1016/S0140-6736(12)60240-2. 22579043

[2] OECD. Does Healthcare Deliver?: Results from the Patient-Reported Indicator Surveys (PaRIS), OECD Publishing, Paris, 2025. 10.1787/c8af05a5-en.

[3] World Health Organization. Framework on integrated, people-centred health services. Geneva: WHO; 2016.

[4] Starfield B, et al. Contribution of primary care to health systems and health. Milbank Q. 2005;833:457–502. 10.1111/j.1468-0009.2005.00409.x. PMC2690145 16202000

[5] Hayes SL, Salzberg CA, McCarthy D, Radley DC, Abrams MK, Shah T, Anderson GF. High-Need, High-Cost Patients: Who Are They and How Do They Use Health Care? A Population-Based Comparison of Demographics, Health Care Use, and Expenditures. Issue Brief (Commonw Fund). 2016;26:1-14. PMID: 27571599. 27571599

[6] Haggerty JL, et al. Continuity of care: a multidisciplinary review. BMJ. 2003;327:1219–21. 10.1136/bmj.327.7425.1219. PMC274066 14630762

[7] Aarhus University. Master’s Programme in Advanced Clinical Nursing. Aarhus: Aarhus University. 2019. Available from: https://ph.au.dk/research/research-areas/clinical-public-health/nursing-and-healthcare

[8] Frederiksen K, Norlyk A, Søndergaard SF. Avanceret Sygeplejepraksis i kommunerne – en Videnskabelig evaluering. Aarhus: Aarhus University & VIA University College; 2024.

[9] Søndergaard SF, Andersen AB, Frederiksen K. APN nurses' core competencies for general clinical health assessment in primary health care. A scoping review. Scandinavian Journal of Caring Sciences. 2024. 10.1111/scs.13235. 38246856

[10] Søndergaard SF, Frederiksen K, Norlyk A. Advanced practice nursing in Danish municipalities: a scientific evaluation. Aarhus: Aarhus University; 2025. Available from: https://sygeplejevidenskab.dk/wp-content/uploads/2025/03/APN-sygeplejepraksis-i-kommunerne-en-videnskablig-evaluering.pdf

[11] Gerrish K, McDonnell A, Nolan M, Guillaume L, Kirshbaum M, Tod A. The role of advanced practice nurses in knowledge brokering as a means of promoting evidence-based practice among clinical nurses. J Adv Nurs. 2011;67(9):2004–14. 10.1111/j.1365-2648.2011.05642.x. 21507046

[12] Lindblad E, et al. Advanced practice nurses’ experiences of the new role in Swedish primary health care: a qualitative study. Int J Nurs Pract. 2010;16:69–74. 10.1111/j.1440-172X.2009.01800.x. 20158551

[13] Ljungbeck B, Sjögren Forss K. Advanced nurse practitioners in municipal healthcare as a way to meet the growing healthcare needs of the frail elderly: a qualitative interview study with managers, doctors and specialist nurses. BMC Nurs. 2017;16:63. 10.1186/s12912-017-0258-7. PMID: 29176932; PMCID: PMC5689167. PMC5689167 29176932

[14] Fagerström L. The impact of advanced practice nursing in healthcare: recipe for developing countries. Ann Neurosci. 2012;19(1):1-2. 10.5214/ans.0972.7531.180401. PMID: 25205953; PMCID: PMC4117071. PMC4117071 25205953

[15] Furlong E, Smith R. Advanced nursing practice: policy, education and role development. J Clin Nurs. 2005;14(9):1059-66. 10.1111/j.1365-2702.2005.01220.x. PMID: 16164523. 16164523

[16] Sulosaari V, et al. Advanced practice nursing education in nordic and Baltic countries. Nord J Nurs Res. 2023;431:14–22. 10.1177/20571585221118891.

[17] Pulcini J, et al. An international survey on advanced practice nursing education, practice, and regulation. J Nurs Scholarsh. 2010;421:31–9. 10.1111/j.1547-5069.2009.01322.x. 20487184

[18] Delamaire ML, Lafortune G. Nurses in advanced roles: a description and evaluation of experiences in 12 developed countries. OECD Health Working Papers No. 54. Paris: OECD Publishing; 2010. 10.1787/5kmbrcfms5g7-en

[19] Kilpatrick K, et al. A global perspective of advanced practice nursing research: a review of systematic reviews. PLoS ONE. 2024;197:e0305008. 10.1371/journal.pone.0305008. PMC11218965 38954675

[20] World Health Organization. Primary health care: fact sheet. Geneva: WHO; 2025.

[21] Winpenny E, Miani C, Pitchforth E, et al. Moving care from hospital into the community: International experiences (substudy 5). I Outpatient services and primary care: Scoping review, substudies and international comparisons (Health Services and Delivery Research). NIHR Journals Library. 2016;4(15):7. https://www.ncbi.nlm.nih.gov/books/NBK361199/. 27195361

[22] Dufour E, et al. Examining nursing processes in primary care settings using the chronic care model: an umbrella review. BMC Prim Care. 2023;24:176. 10.1186/s12875-023-02089-3. PMC10476383 37661248

[23] Bales G, Hasemann W, Kressig RW, Mayer H. Scope of practice, competencies and impact of advanced practice nurses within APN-led models of care for Multimorbidity and complex chronic conditions in hospitals: a scoping review. BMJ Open. 2025;15:e091170. 10.1136/bmjopen-2024-091170. PMC12039040 40295123

[24] OECD. OECD Reviews of Health Care Quality. Primary care in Denmark. Paris: OECD Publishing; 2017. 10.1787/9789264269453-en.

[25] Wagner EH, Austin BT, Von Korff M. Organizing care for patients with chronic illness. Milbank Q. 1996;74(4):511-44. PMID: 8941260. 8941260

[26] World Health Organization. Continuity and coordination of care. Geneva: WHO; 2018.

[27] Valentijn PP, et al. Understanding integrated care. Int J Integr Care. 2013;13:e010. 10.5334/ijic.886. PMC3653278 23687482

[28] Yin RK. Case study research: design and methods. 5th ed. Thousand Oaks [CA]: SAGE; 2014.

[29] Olsen H. Kvalitative analyser og kvalitetssikring. Sociologisk Forskning. 2003;40(1):68–103. 10.37062/sf.40.19395.

[30] Pawson R, Tilley N. Realistic evaluation. London: SAGE; 1997.

[31] Nielsen P. Produktion Af viden. 3. udg. København: Nyt Teknisk Forlag; 2009.

[32] Petersen KS, et al. Udvikling, test, evaluering Og implementering. Aalborg: Aalborg Universitetsforlag; 2022.

[33] Rockwood K, et al. Clinical frailty Scale – 9 point scale. Halifax: Dalhousie University; 2020.

[34] Vinge S. Kompleksitet i den kommunale sygepleje – En analyse af sygeplejerskernes perspektiver på kompleksitet i sygeplejen. København: VIVE – Det Nationale Forsknings- og Analysecenter for Velfærd; 2018. Available from: https://www.vive.dk/da/udgivelser/kompleksitet-i-den-kommunale-sygepleje-rv73bdxn/

[35] Brinkmann S, Interviews. Learning the craft of qualitative research interviewing. 3rd ed. Thousand Oaks [CA]: Sage; 2015.

[36] Braun V, Clarke V. Using thematic analysis in psychology. Qual Res Psychol. 2006;32:77–101. 10.1191/1478088706qp063oa.

[37] Fox S, McAllum K, Mikkola L, editors. Interprofessional communication in health and social care. Cham: Springer; 2025. 10.1007/978-3-031-70106-1.

[38] Eckerblad J, Theander K, Ekdahl AW, Unosson M, Krevers B. Symptom burden in community-dwelling older people with multimorbidity: a cross-sectional study. BMC Geriatr. 2015;15:1. 10.1186/1471-2318-15-1. PMC4292813 25559550

[39] Nutbeam D. Health literacy as a public health goal: a challenge for contemporary health education and communication strategies into the 21st century. Health Promot Int. 2000;15[3]:259 – 67. 10.1093/heapro/15.3.259

[40] Sørensen K, Van den Broucke S, Fullam J, Doyle G, Pelikan J, Slonska Z, et al. Health literacy and public health: a systematic review and integration of definitions and models. BMC Public Health. 2012;12:80. 10.1186/1471-2458-12-80. PMC3292515 22276600

[41] Kitson A, Conroy T, Wengström Y, Profetto-McGrath J, Robertson-Malt S. Defining the fundamentals of care. Int J Nurs Pract. 2010;164:423–34. 10.1111/j.1440-172X.2010.01861.x. 20649678

[42] Feo R, Rasmussen P, Wiechula R, Conroy T, Kitson A. Developing effective and caring nurse-patient relationships. Nurs Stand. 2017;31(28):54-63. 10.7748/ns.2017.e10735. PMID: 28271761. 28271761

