# Supplementary material for: The value of advanced practice nursing in Danish primary care: a case study of communicative and integrative mechanisms in community-based care
Source: BMC Prim Care. 2026 Feb 6;27:90. doi: 10.1186/s12875-026-03207-7 (PMC12977751; doi:10.1186/s12875-026-03207-7)
Supplement: Supplementary file 1 — Supplementary Material 1 [file 12875_2026_3207_MOESM1_ESM.docx]

Additional file 1

All participants received both oral and written information about the purpose, content, and format of the interview. At the start of each interview, participants were reminded that the interview was open-ended, that there were no right or wrong answers, and that they were encouraged to speak freely. All participants provided written informed consent prior to participation.

Interview- and workshop guides

[A. Interview Guide for Citizens and Relatives 2](#_Toc214106262)

[B. Interview Guide for Municipal Collaborators 4](#_Toc214106263)

[C. Interview Guide for General Practitioners 5](#_Toc214106264)

[D. Semi-Structured Guide for the Reflective Workshop with Advanced Practice Nurses 6](#_Toc214106265)

[E. Information Sheet – Workshop on Advanced Practice Nursing (APN) 8](#_Toc214106266)

# Interview Guide for Citizens and Relatives

The original guide was developed in Danish and subsequently translated into English for the purpose of this publication.

#### Theme: Experiences with Advanced Practice Nurses (APNs) in Primary and Community Care

## Introductory Questions

- Could you start by telling me a bit about your situation and the background for your contact with the APN?
- How did your first meeting with the APN take place, and what do you remember most clearly from it?

## Experience of the APN’s Role and Presence

- How would you describe your experience of the APN’s involvement in your (or your relative’s) care?
- What stands out to you about the APN’s way of working or being present?
- How did the APN’s presence influence how you felt in the situation?

## Communication and Relationship

- How would you describe the communication between you and the APN?
- In what ways did the APN help you feel informed, understood, or supported?
- Were there any specific moments, conversations, or small actions that made a difference to you?

## Coordination and Continuity

- How did you experience the coherence of the care pathway while the APN was involved?
- Did you notice anything about how the APN collaborated with other professionals?
- In what ways did the APN contribute to creating continuity or an overview of the process?

## Impact on Your Situation or Everyday Life

- How did the APN’s involvement affect your overall experience of the care pathway?
- Did the APN help you feel more secure, supported, or prepared?
- Looking back, what difference do you think the APN made for you or your relative?

## Needs, Preferences, and Improvements

- Was there anything you would have wished for more of, or done differently?
- What could strengthen the experience for citizens and relatives in similar situations?
- What advice would you give to healthcare professionals about what matters most to you?

## Closing Questions

- Is there anything we have not talked about that you think is important to mention?
- Is there a particular experience with the APN that you would like to highlight?

# Interview Guide for Municipal Collaborators

The original guide was developed in Danish and subsequently translated into English for the purpose of this publication.

**Theme: Collaboration with Advanced Practice Nurses (APNs) in Primary and Community Care**

Participants: Registered Nurses, Nursing Assistants, and Home Care Staff

## Experience and Contact

- How would you describe your role, and in what ways do you interact with APNs?
- How do you experience their presence and function in your everyday practice?

## Systematic Work and Methods

- How would you describe the way APNs work within primary and community care?
- What methods, principles, or tools do you observe APNs using in their practice?
- In what ways does the work of APNs influence your own tasks or decision-making?

## Coordination and Holistic Approach

- How do you experience the APNs’ role in ensuring coherence and coordination in the citizen’s care pathway?
- How would you describe the APNs’ approach to holistic assessment and early identification of needs or deterioration?

## Collaborative Relationships

- How would you describe the collaboration between the APNs and your team?
- What aspects of the collaboration work particularly well, and where do you see room for improvement?

## Impact and Suggestions

- What contributions do you experience APNs making to citizen care or team functioning?
- What suggestions do you have for strengthening the collaboration going forward?

## Closing Questions

- Is there anything we have not talked about that you think is important to mention?
- Is there a particular experience with the APN that you would like to highlight?

# Interview Guide for General Practitioners

The original guide was developed in Danish and subsequently translated into English for the purpose of this publication.

**Theme: Collaboration with Advanced Practice Nurses (APNs) in Aalborg Municipality**

## Introductory Questions

- How would you describe your experiences with APNs in the municipality?
- In what situations do you typically come into contact with APNs, and how does this contact unfold?

## Collaboration and Coordination

- How would you describe how the collaboration functions in your daily practice?
- Which types of patients do you find yourselves collaborating on, and how does this collaboration emerge?
- In what ways do you experience APNs working systematically or in a coordinating role?

## Communication and Knowledge Sharing

- How would you describe the communication between you and the APNs?
- What tools, routines, or structures do you experience as supporting the collaboration?

## Value and Impact

- In what ways do you experience APNs contributing to patient care or workflow?
- How, if at all, have you noticed changes in patient pathways as a result of working with APNs?

## Barriers and Opportunities for Improvement

- What challenges, if any, have you experienced in the collaboration?
- How do you think the collaboration could be strengthened or improved?

## Closing Questions

- Is there anything we have not talked about that you think is important to mention?
- Is there a particular experience with the APN that you would like to highlight?

# Semi-Structured Guide for the Reflective Workshop with Advanced Practice Nurses

The original guide was developed in Danish and subsequently translated into English for the purpose of this publication.

## Purpose

To facilitate a structured reflection on empirical findings from interviews with patients, relatives, municipal collaborators, and general practitioners regarding Advanced Practice Nursing in Danish primary care.

## Section 1. Thematic Reflection Questions

### Theme 1: Holistic and Analytical Approach to Complex Care

Prompting quotes:

- “An APN can do some of the same things as a doctor—not everything, of course, but her perspective on the citizen is more holistic…”
- “She traces the patient’s history far back and identifies connections and unresolved issues…”

Guiding questions:

1. How do these findings reflect your own practice?

2. What actions do you take to ensure coherence and continuity in complex care pathways?

### Theme 2: Collaboration and Professional Partnership

Prompting quotes:

- “I had a partnership with this APN that was incredibly valuable to me…”
- “That partnership meant I did not give up—it was a relief and a major professional support…”

Guiding questions:

1. How do you experience collaboration with general practitioners and other professionals?

2. How do you enact such partnerships in your daily work?

### Theme 3: Relational Continuity and Sense of Security

Prompting quotes:

- “You felt safe in everything when she was there.” (Relative)
- “It mattered that she was the one following me—it made me feel safe.” (Citizen)

Guiding questions:

1. How do you actively create relational continuity and a sense of security for patients and relatives?

2. Which specific actions or “micro-actions” contribute most to patients feeling seen and safe?

### Theme 4: Tacit Knowledge and Invisible Work

Guiding questions:

1. Which aspects of your work are typically invisible to others?

2. How does your APN training support the competencies you apply in practice?

## Section 2. Manager Perspective

Questions for the manager:

1. How do you observe the APN role unfolding in practice?

2. In which areas do APNs contribute distinct value within the organisation?

Follow-up question for APNs:

- Would you like to add or nuance anything based on the manager’s comments?

## Section 3. Closing Reflection

Final question:

- What is the most important insight you take away from the workshop?

1. Information Sheet – Workshop on Advanced Practice Nursing (APN)

*The original information sheet was developed in Danish and subsequently translated into English for the purpose of this publication.*

The information sheet was emailed to the participating APN and their manager 2 weeks before the workshop.

**Purpose**

The workshop is part of the research project on the value of Advanced Practice Nurses in Danish primary care. The purpose is to present findings from interviews with patients, relatives, municipal collaborators, and general practitioners, and together with you refine and deepen the understanding of APN work. We aim in particular to make visible the competencies and actions that are often invisible from the outside but are essential to your practice.

**Process**

The workshop lasts 1.5 hours and is conducted as a collective reflection. Selected quotes and themes from the interview material will be presented as a basis for dialogue. We will work in plenary and ensure space for everyone’s perspectives.

**Programme**

1. Introduction and framing
2. Presentation of empirical findings
3. Reflective dialogue on key themes
4. Manager’s perspective
5. Summary and closing

**Practical Information**

- Duration: 1.5 hours
- Participants: 6 APN nurses and your direct manager
- Location: Anneshave temporary care unit
- Time: 19th. September 2025, 09:30–11:00

All contributions will be treated confidentially and anonymized in the subsequent analysis. Participation is voluntary, and the purpose is to co-create new knowledge, not to evaluate your work.
